# Characterizing Emotional State Transitions During Prolonged Use of a Mindfulness and Meditation App: Observational Study

**DOI:** 10.2196/19832

**Published:** 2021-03-02

**Authors:** Argus Athanas, Jamison McCorrison, Julie Campistron, Nick Bender, Jamie Price, Susan Smalley, Nicholas J Schork

**Affiliations:** 1 Bioinformatics and Systems Biology University California San Diego San Diego, CA United States; 2 Stop, Breathe & Think, Inc Los Angeles, CA United States; 3 University California Los Angeles Los Angeles, CA United States; 4 Department of Quantitative Medicine, The Translational Genomics Research Institute An Affiliate of the City of Hope National Medical Center Phoenix, AZ United States; 5 The City of Hope/Translational Genomics Research Institute IMPACT Center Duarte, CA United States

**Keywords:** mental health, mobile apps, smartphone, mobile phone, emotional distress, mindfulness

## Abstract

**Background:**

The increasing demand for mental health care, a lack of mental health care providers, and unequal access to mental health care services have created a need for innovative approaches to mental health care. Digital device apps, including digital therapeutics, that provide recommendations and feedback for dealing with stress, depression, and other mental health issues can be used to adjust mood and ultimately show promise to help meet this demand. In addition, the recommendations delivered through such apps can also be tailored to an individual’s needs (ie, personalized) and thereby potentially provide greater benefits than traditional “one-size-fits-all” recommendations.

**Objective:**

This study aims to characterize individual transitions from one emotional state to another during the prolonged use of a digital app designed to provide a user with guided meditations based on their initial, potentially negative, emotional state. Understanding the factors that mediate such transitions can lead to improved recommendations for specific mindfulness and meditation interventions or activities (MMAs) provided in mental health apps.

**Methods:**

We analyzed data collected during the use of the Stop, Breathe & Think (SBT) mindfulness app. The SBT app prompts users to input their emotional state before and immediately after engaging with MMAs recommended by the app. Data were collected from more than 650,000 SBT users engaging in nearly 5 million MMAs. We limited the scope of our analysis to users with 10 or more MMA sessions that included at least 6 basal emotional state evaluations. Using clustering techniques, we grouped emotions recorded by individual users and then applied longitudinal mixed effect models to assess the associations between individual recommended MMAs and transitions from one group of emotions to another.

**Results:**

We found that basal emotional states have a strong influence on transitions from one emotional state to another after MMA engagement. We also found that different MMAs impact these transitions, and many were effective in eliciting a healthy transition but only under certain conditions. In addition, we observed gender and age effects on these transitions.

**Conclusions:**

We found that the initial emotional state of an SBT app user determines the type of SBT MMAs that will have a favorable effect on their transition from one emotional state to another. Our results have implications for the design and use of guided mental health recommendations for digital device apps.

## Introduction

### Background

The motivation for managing mental health disorders, precursors of mental health disorders, and emotional problems generally, on a footing equal to how physical health disorders are managed, is growing [1-3]. Mental health disorders, stress, anxiety, depression, and other mood-related conditions are known to affect productivity, comorbid conditions, and physical well-being [4-6]. In fact, when asked, approximately 90% of Americans stated that they value mental health as much as they value physical health [7]. This is not without reason, as the prevalence of anxiety disorders alone in the population at large is estimated to be between 3.8% and 25.0% [8]. Given the high collective prevalence of mental health concerns, behavioral conditions, and mood-related maladies, their impact on quality of life, and the costs associated with the care of individuals affected by such conditions, there is a great need to develop more efficient and reliable ways of not only treating these problems but also preventing them. Unfortunately, developing an appropriate infrastructure to combat mental health problems within the current health system will be daunting and expensive, as many people find available mental health care overly complicated and often inaccessible [9-12]. Fortunately, newer and more accessible approaches to the care of individuals with mental health issues are being developed, including the use of telehealth, an emphasis on risk mitigation as opposed to treatments, and the use of digital therapeutics [13]. Of these, digital therapeutics are receiving significant attention. Digital therapeutics are programs (eg, smartphone apps) that provide guidance on stress or symptom management and alleviation, or to qualitatively change an individual’s mood or emotional state in some way (eg, via imagery, mood rating, or tracking), and can be used remotely and at an individual’s discretion.

The great potential of digital therapeutics is recognized by public health and government regulatory agencies as well. The Food and Drug Administration has allowed mobile mental health apps to receive approvals and accreditation as bona fide medical health interventions on a similar footing with drugs [14-17]. Care that includes the use of mental health apps can be scaled to help meet the demand for mental health care because the use of these apps may not require as much interaction with trained professionals, and standard clinical exams associated with pharmacotherapy monitoring might also require travel and interaction with a health care provider (eg, for dosing or evaluating potential physical side effects). More importantly, mental health apps have great potential to help provide care in underserved populations where financial, professional scarcity, and societal burdens make other forms of care problematic [18]. Mental health apps have obvious limitations as they are not appropriate for use in all settings. One example of where their use is appropriate is in behavioral and mental health settings involving stress management, where techniques such as encouraging relaxation via, for example, meditations and mindfulness, can be used. In fact, mindfulness and meditation activities (MMAs) have been linked with healthy thought patterns and improved mood and can reduce stress and anxiety that are often precursors or symptoms of certain mental health concerns [19,20].

Unfortunately, although the promise of mental health apps for reducing the risk of and treating some mental health issues and concerns are great, there is a need to vet different strategies for creating mental health apps and understanding the settings in which they might be most effective. This is partly due to difficulties in defining mental health concerns and tracking individuals’ symptoms over time in a way that can shed light on when to intervene and in what manner. This is true for very serious mental health disorders, such as treatment-resistant depression, as it is for managing daily stress and anxiety that, if prolonged, could lead to more dramatic mental health issues. For example, determining which personal settings and emotional states are appropriate for different interventions, such as MMAs, are yet to be explored in full [21]. In fact, it is quite likely that there exists a great deal of intra- and interindividual variability in mood and feelings of stress and anxiety that might be necessary to understand and characterize so that guidelines and interventions, such as MMAs, can be tailored or personalized to individual users.

### Objectives

We pursued a series of analyses to explore how the moods of the users of the SBT MMA app changed or transitioned as they used the app and its MMA recommendations. As discussed in our previously published paper on data collected via the SBT MMA app [22], the SBT app recommends sets of MMAs to users based on their mood at the time they use the app. In our previous work, we observed a statistically significant trend for improvement in basal mood (eg, a shift toward moods consistent with happiness or less anger) with prolonged use of the app. It was found that most users’ moods tended to improve after a single session with an MMA recommended by the app.

In the studies described here, we assessed the specific associations that the recommended MMAs have with the transitions between moods of users at baseline and after they participate in a recommended MMA over the period in which they use the app. In this light, our previous studies motivated the present work, as we did not study the specific transitions from one emotional or mood state to another in the original work; that is, our previous finding that the SBT app did lead to acute and chronic changes in mood, mostly for the better, led to our interest in determining which specific mood states are associated with the use of the app and the specific MMAs. A better understanding of which MMAs are likely to drive changes in emotional states and in which directions could lead to insights into which MMAs might be appropriate for individuals with specific emotional state profiles. Such an understanding could lead to better predictions and MMA recommendations for individual users.

## Methods

### The App

**Figure 1:**
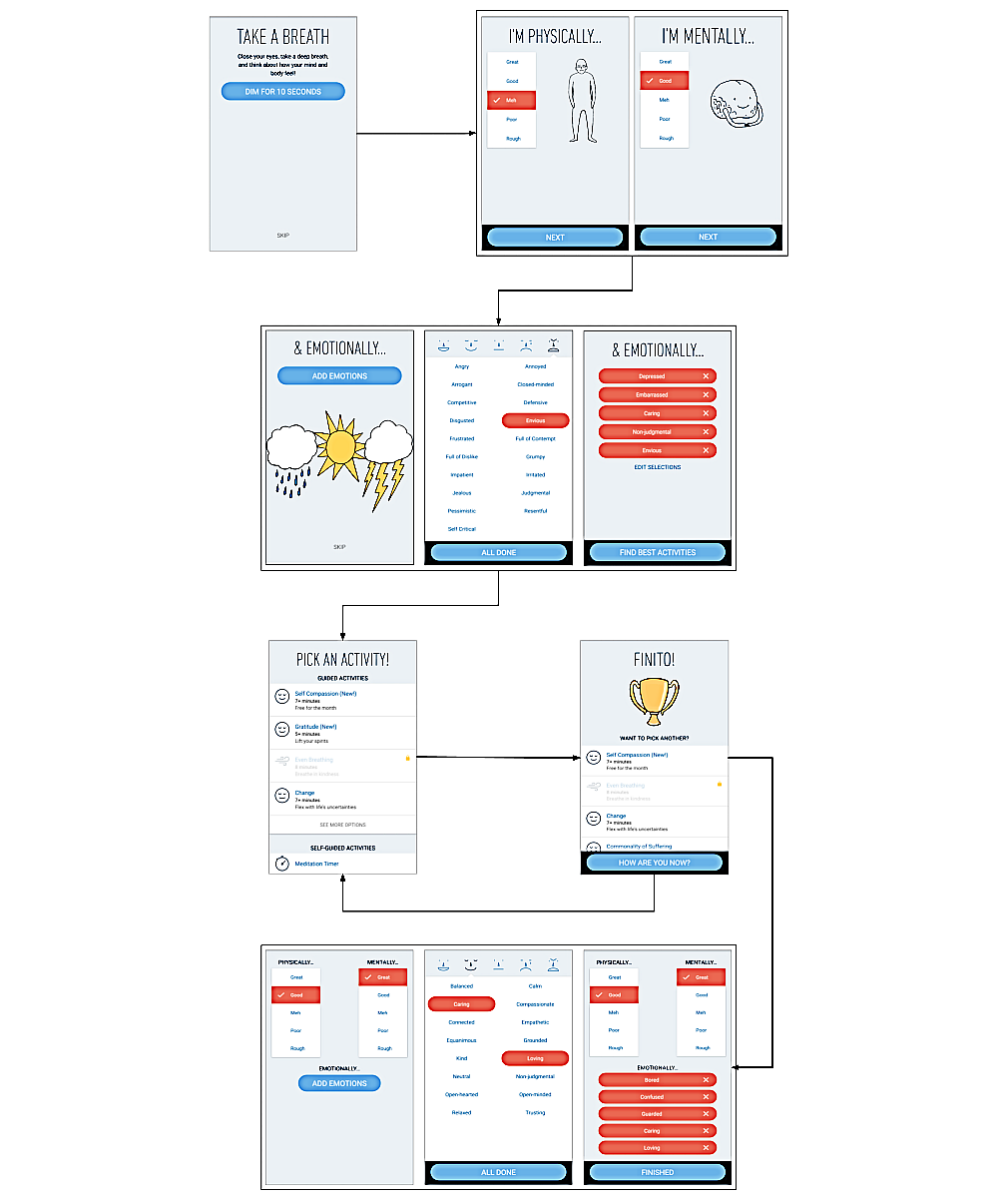
Stop, Breathe & Think (SBT) app user flow diagram. A diagram depicting the users’ experience when engaging the SBT app. A natural flow allows the user to reflect, check-in, perform an activity, and then check-in again. Reflections and check-ins are optional but were required data points for our analysis.

The app developed by SBT is designed to guide users through MMAs, which are created to reduce stress, anxiety, and depression and improve internal focus, mood, mental state, and sleep. The SBT app is designed for general use and can deliver MMAs through many different platforms (ie, iOS, Android, Alexa). When the app is used, a user is prompted to perform an optional 10-second reflection, which is followed by optional prompts to state how they are feeling mentally, physically, and emotionally. The questions used to assess mental and physical status are based on a 5-point scale with the following categories: great, good, meh, poor, and rough. Following this, users are asked to pursue an emotional check-in involving a selection of 1 to 5 terms from a pool of 115 emotions that describe their current emotional state. After this initial check-in, users are shown some suggested MMAs with labels and context designed, for example, for *Gratitude, Silence, Breathing*, but they are free to select an MMA of their preference within a defined set. After the completion of an MMA, users can continue to select additional MMAs or end the session. At the end of each session, users are again prompted to do another optional check-in to assess their mental, physical, and emotional state. A flow diagram of the user experience with the SBT app is shown in Figure 1.

We emphasize that all data collected through the SBT app are volunteered by users as stated in the SBT user licensing agreement and privacy policy, and thus, the characterizations of mood and physical well-being are subjective. All reported data were anonymized and aggregated into a data set reflecting user experiences: usernames were replaced with unlinked hash keys, personal information was stripped, and only reporting of the first 3 numbers of zip codes was maintained. This information was ultimately put into a Health Insurance Portability and Accountability Act–compliant format so that users could not be reidentified.

Our data preparation methods were nearly identical to those described in our previous publication on SBT data. However, in the current analysis, the data from several users were removed from all analyses to adhere to privacy policies and best practices in consultation with the legal and compliance team at SBT. Owing to our deidentification process, we could not distinguish which users were removed from previous analyses or which users are now included because they have since met active user filtering criteria. However, based on information about when users started to use the SBT app, it can be inferred that there were 856 new active users who passed our filtering criteria since our last publication, and at least 3219 users were removed for the analyses described here that were considered in our previous analyses because of the adoption of other filtering criteria at SBT. The SBT app has variation in functionality and delivery across platforms, and to avoid confounding effects, we focused our analysis on iOS users only. Users who joined before the last major update of the app (May 1, 2016) were also excluded. Further filtering was pursued to only include active users with ≥10 sessions completed, who had at least six sessions in which they provided both pre- and postemotional check-in information. To avoid cultural differences and language barriers, only users from English-speaking countries (United States, United Kingdom, Canada, and Australia) were included. Finally, for both compliance reasons as well as concerns about the accuracy of the information, we excluded users aged below 13 years or above 100 years from the analysis.

### Clustering Emotions

As described in our previous paper, emotions endorsed by users of the app were grouped into clusters based on the user's selection of multiple emotional terms to characterize their complete emotional state at that particular time. Emotions were compared using the Bray-Curtis dissimilarity according to how often they were coselected [23]. We used Principal Coordinate Analysis (PCoA) to translate emotion endorsement dissimilarity into two-dimensional space using the first 2 PCoAs, and then, partitioning around medoids (PAM) was used along with silhouette scores to determine the optimal number of clusters [24]. Each of the 115 emotions was then assigned to a cluster, and the corresponding cluster medoid was recorded. Individual emotional states, both pre- and post-MMA engagement, were defined by the nearest cluster (in terms of Euclidean distance). These clusters define distinct emotional state categories, and in the current analyses, we focused on the change (or transition) in emotional state categories between pre-MMA and post-MMA.

**Figure 2:**
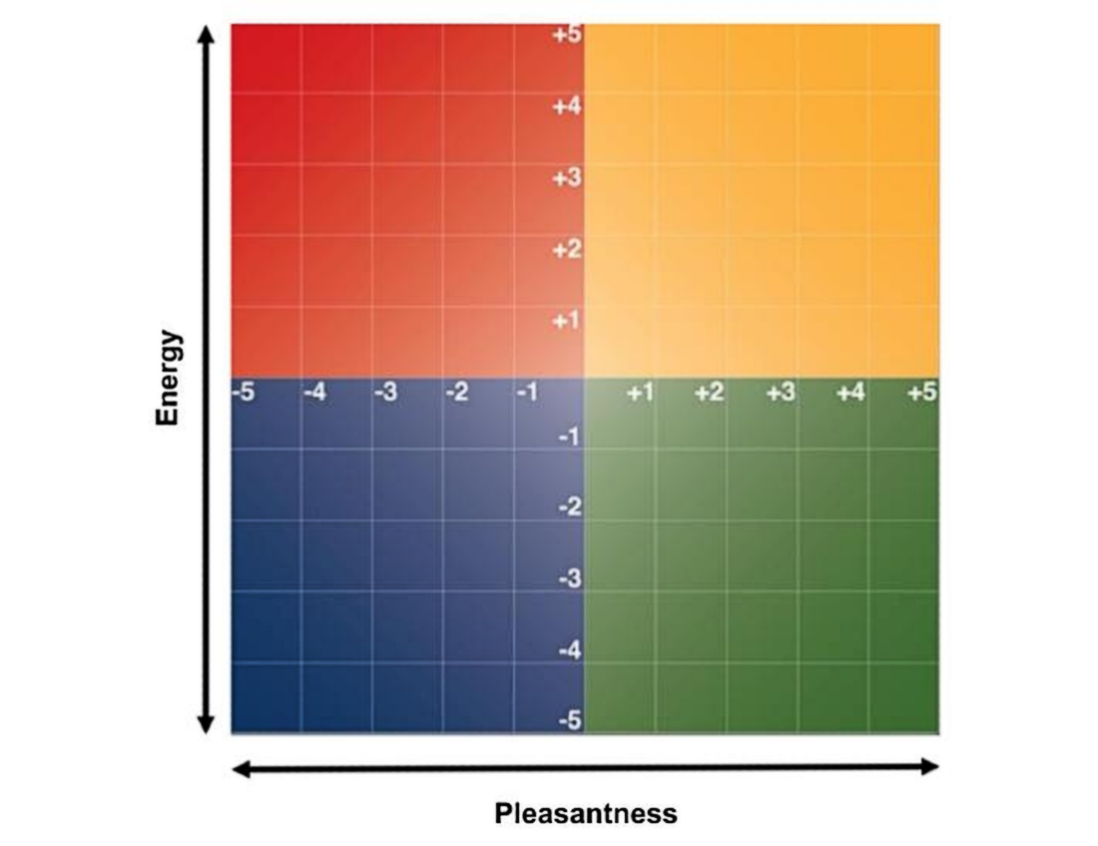
Yale Mood Meter (see the main text for a description and references). A 2 dimensional framework which classifies emotions by their pleasantness (x-axis) and energy (y-axis). Yellow and green quadrants are favorable states whereas red and blue quadrants are unfavorable.

To better understand and synthesize the results of our emotion clustering, we projected the clusters onto the Yale Mood Meter (YMM; Figure 2) [25]. The YMM groups emotions into 4 quadrants, which are defined by the “energy” of the emotion (y-axis) and the “pleasantness” of the emotion (x-axis). We color-coded these quadrants using the accepted criteria: red=high energy, low pleasantness (denoted as “HELP”); yellow=high energy, high pleasantness (HEHP); blue=low energy, low pleasantness (LELP); and green=low energy, high pleasantness (LEHP). We assigned each of the clusters to one of the 4 quadrants based on the majority of emotions within each cluster that mapped to a YMM quadrant. This mapping allows us to consider transitions from quadrant to quadrant instead of simply a cluster-to-cluster membership, which has several advantages: (1) it reduces our search space from 64 transitions to 16; (2) it increases the sample size for each transition, thus providing better power to detect changes; and (3) it provides an interpretable scale for transitions (ie, an HELP state [red] to HEHP state [yellow]). Although users may have different objectives in engaging the app and an MMA, the assumption is that red (HELP) and blue (LELP) states are undesirable, whereas yellow (HEHP) and green (LEHP) are desirable.

A framework for categorizing emotions developed by Brackett [25] classifies emotions into a two-dimensional space with pleasantness as the x-axis and energy as the y-axis. The negative emotion quadrants red and blue represent low pleasantness, red is higher energy such as anger, and blue is low energy such as sadness. The more favorable quadrants, green and yellow, represent high pleasantness, with energetic emotions such as “excited” fitting in the yellow quadrant, and lower energy emotions such as “calm” fitting in the green quadrant.

### Description of the SBT MMAs

The SBT app provides over 100 different MMAs for users to choose from, with varying levels of popularity and usage. Given the number of MMAs and the risk of overfitting in our analysis models, we first considered each MMA individually and then ultimately focused on the top 20 chosen MMAs (representing 86.8% of all completed MMAs) and combined the rest (a total of 21 MMA categories assessed in our analyses) into a single category called “other.” The frequency distribution of these MMAs and their descriptions can be found in Multimedia Appendix 1.

### Statistical Analysis

To determine the strength of the association between each of the 20 most chosen MMAs and the “other” MMA category and the transition from one emotional state to another, we used generalized linear models (GLMs) as implemented in the R package lme4 [26]. GLMs have many features that make them appropriate for our analyses. For example, GLMs can accommodate and quantify serial correlations among variables in longitudinal analyses, and both fixed and random effects can be considered as important covariates. Random effects are important to consider because they can account for variation in moods chosen by individual SBT users attributable to unmeasured covariates such as individual-specific effects of unknown origin (eg, personal habits, unmeasured stressors, or exposures, etc). GLMs have also been widely used in the statistical analysis of many psychiatric and psychological phenomena [27-29]. For each YMM quadrant, we determined which users’ moods were associated with that quadrant when they initially used the app. We then fit a logit-link GLM to the data to test the associations between the basal mood quadrant of a user and the quadrants that the user transitioned to after engaging in the MMA. To enable this, we created dummy variables to indicate whether a user transitioned to a specific YMM quadrant and used this as a dependent variable in the GLM with basal YMM quadrant, count data for the number of MMAs a user completed in a session, age, sex, session index (ie, 1 as the first use of the application, 2 as the second use, etc), as independent variables. We also included other covariates, such as subscription status, user account completion, and time between sessions as additional independent variables. We used the country code in the initial analyses but excluded it in subsequent analyses due to its statistical insignificance. Due to the differences in the number of user engagements (the range was from 10 uses to 1044 uses), we used a log_10_ transform on session index, which is consistent with our strategy in our previous analysis of the SBT data. All non-MMA independent variables were standardized so that the resulting model beta values (ie, standardized regression coefficients) could be directly compared.

### Building a Prototype Learning System for Predicting Emotion Transitions

To evaluate the effectiveness of our analytical models in predicting transitions, we used GLM analysis but restricted the data to a “training set” consisting of 3 sequential observations per user starting with their first completed recorded session. After obtaining models for each of the possible transitions based on data from these 3 initial sessions, we used the models to predict the probability of each transition in subsequent sessions and then selected the transition with the highest probability and matched it with the observed transitions. This allowed us to compare the actual emotional state transition with a predicted transition state and to see if the app could be improved by anticipating MMAs likely to result in positive mood transition in real time. We repeated this analysis several times using both different numbers of initial engagements with the app and the time intervals in our training sets to further evaluate its performance.

## Results

### Data Set Summary

Before any filtering, we had observations for nearly 5 million engagements with SBT app MMAs across 677,000 different users. There were 84,000 active users who completed 10 or more sessions, who collectively completed 3.16 million engagements with MMAs. After filtering for the operating system, which users were active, language used in the app, and quality, 11,030 unique users were included in subsequent analyses. These users collectively completed 289,360 MMAs across 253,363 sessions (average of 1.14, SD 0.51 MMAs per session). As shown in Multimedia Appendix 2**,** most users were female (8274/11,030, 75.01%) and were between the ages of 13 and 40. Compared with our previous study, we had fewer users and sessions, due to their removal for legal and compliance reasons, but the users whose data we possessed completed more sessions and emotional check-ins on average.

### Clustering Analysis of Emotions

**Figure 3:**
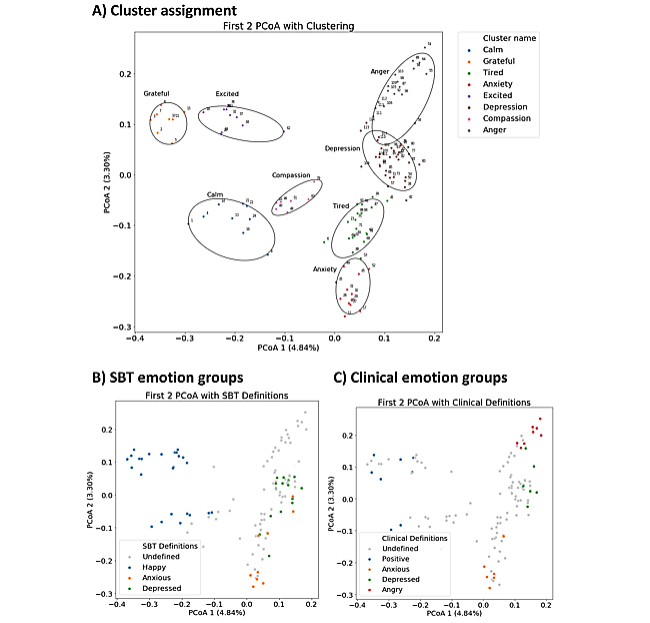
Emotional clustering on co-selected terms. Emotional clusters created from coselected terms within the same check-in (see text), are defined using PAM and silhouette scores. Each point represents an emotion that can be endorsed, and can be looked up in corresponding table. (A) The optimal 8 clusters are shown across the first 2 principal coordinate analyses (PCoAs). Clusters are given labels based on a single emotion that is thematic to most emotions within the cluster. (B) In-house, defined emotional labels show consistent grouping within the first 2 PCoAs. (C) Clinically defined emotional labels show consistent grouping within the first 2 PCoAs.

The results of our cluster analysis of emotions were similar to those described in our previously published paper. The optimal number of clusters as defined by the silhouette scores on the PCoA and PAM analyses was 8. Of the 115 emotions, all but 3 emotions (Envious, Fiery, and Self Critical) were assigned to the same clusters as in our previously published analyses. The 8 clusters grouped emotions into categories with very common themes and were validated with prior SBT internal product and clinical groupings (Figure 3). On the basis of these clusters, from each user’s emotional state pre- and post-MMA, we could determine which category their emotions were most closely associated with using distances of the emotions to cluster medoids.

**Figure 4:**
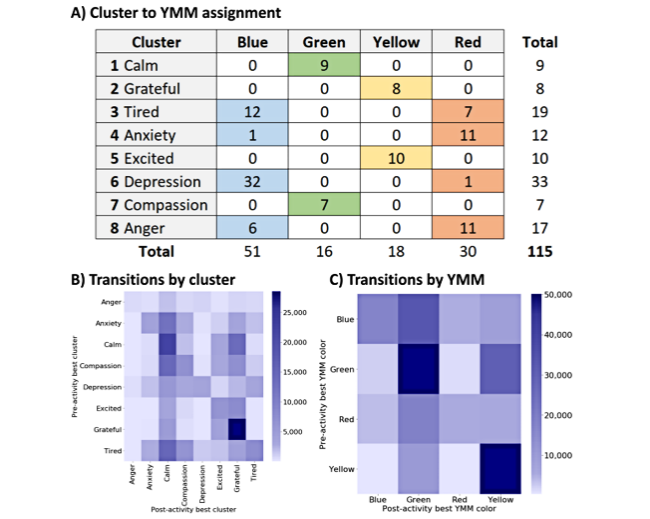
Cluster alignment and pre– to post–mindfulness and meditation activity (MMA) transitions. Clusters were assigned to the Yale Mood Meter (YMM) quadrants based on the majority of emotions within that cluster that corresponded to a YMM quadrant (eg, the “tired” cluster is blue in YMM). (A) Counts of all 115 endorsable emotions in the Stop, Breathe & Think app by each cluster and the YMM quadrant they are associated with. (B) Session counts for transitions from the pre-MMA emotional cluster to the post-MMA emotional state. Calm and grateful clusters were the most frequent post-MMA states. (C) Session counts for transitions from the pre-MMA emotional YMM quadrant to the post-MMA emotional quadrant.

Alignment of clusters to YMM quadrants provided further support for clustering, as most clusters were clearly aligned to a quadrant. As shown in Figure 4, “Calm” and “Compassion” clusters had perfect alignment with the green quadrant, and “Grateful” and “Excited” clusters had perfect alignment with the yellow (HEHP) quadrant. The “Tired” cluster had emotions that crossed between both the blue (LELP; ie, tired, lazy, fatigue) and red (HELP; ie, afraid, panicked, suspicious) quadrants. The “Anger” cluster also had some crossover between red (HELP; ie, angry, impatient, resentful) and blue (LELP; ie, defensive, disgusted, pessimistic) quadrants. Conveniently, the clusters were mapped to quadrants in pairs.

Figure 4 shows the relative frequencies of pre-MMA emotional states and post-MMA emotional states of the users. The most frequent post-MMA cluster was calm, followed by grateful. Users predominately started and ended in positive states (ie, green or yellow clusters), with green (LEHP) being the predominant emotional state that users transitioned to. The most common negative states were tired and anxiety.

### Model Features

**Figure 5:**
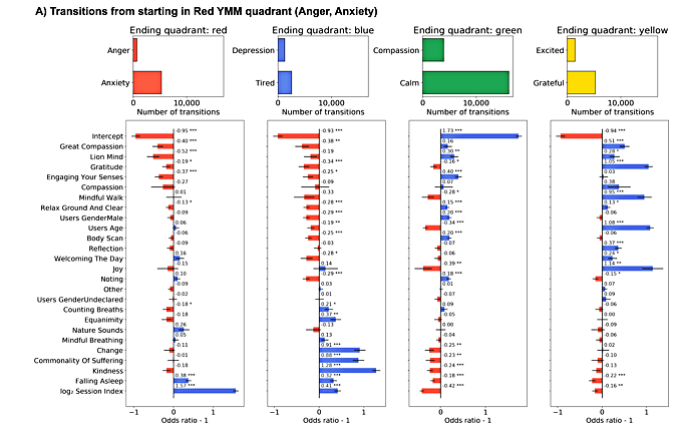
Transitions starting in Red Yale Mood Meter (YMM) quadrant model odds ratios. Sixteen GLM model fixed effect estimated odd ratios and *P* values (<.001***, <.01**, and <.05*) for mindfulness and meditation activities, gender, session index, and y-intercept split by starting YMM quadrant. Blue bars indicate increased propensity to make a transition, whereas red bars indicate decreased probability of making the transition. Transitions starting in Red YMM quadrant, where users who completed the MMA Lion Mind were 52% less likely to stay in Red, 30% more likely to enter Green, and 28% more likely to enter Yellow than those who did not complete Lion Mind.

**Figure 6:**
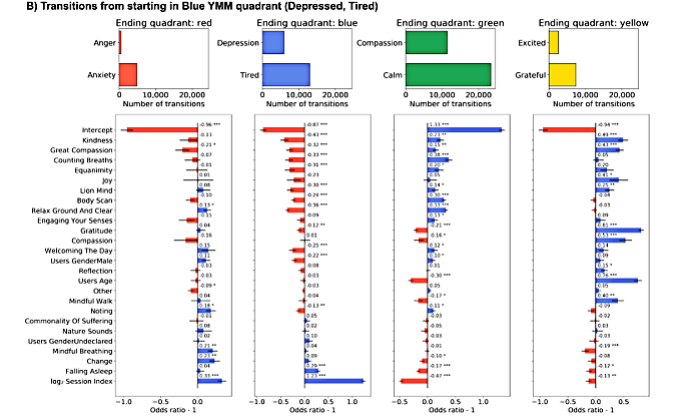
Transitions starting in Blue Yale Mood Meter (YMM) quadrant model odds ratios. Sixteen GLM model fixed effect estimated odd ratios and *P* values (<.001***, <.01**, and <.05*) for mindfulness and meditation activities, gender, session index, and y-intercept split by starting YMM quadrant. Blue bars indicate increased propensity to make a transition, whereas red bars indicate decreased probability of making the transition.

**Figure 7:**
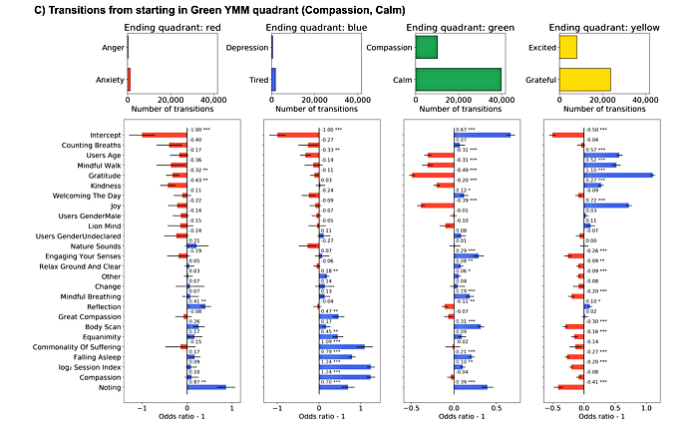
Transitions starting in Green Yale Mood Meter (YMM) quadrant model odds ratios. Sixteen GLM model fixed effect estimated odd ratios and *P* values (<.001***, <.01**, and <.05*) for mindfulness and meditation activities, gender, session index, and y-intercept split by starting YMM quadrant. Blue bars indicate increased propensity to make a transition, whereas red bars indicate decreased probability of making the transition.

**Figure 8:**
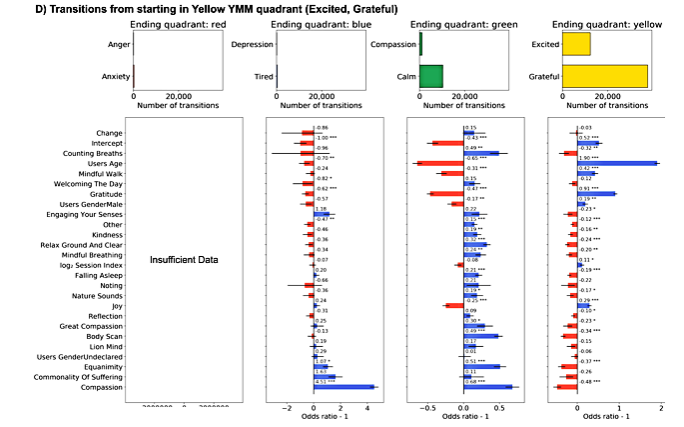
Transitions starting in Yellow Yale Mood Meter (YMM) quadrant model odds ratios. Sixteen generalized linear model fixed effect estimated odd ratios and *P* values (<.001***, <.01**, and <.05*) for mindfulness and meditation activities, gender, session index, and y-intercept split by starting YMM quadrant. Blue bars indicate increased propensity to make a transition, whereas red bars indicate decreased probability of making the transition. There was not enough data to calculate estimates with confidence for transitions from Yellow (HEHP) to Red (HELP).

We did not find any obvious evidence suggesting that a specific MMA is the most effective in transitioning users’ emotions to different categories. Each of the 16 GLMs we fit to the data focused on the influence or association of the different MMAs with different transitions and they exhibit varying degrees of association strength between pre- and post-MMA emotions (Figures 5-8). The MMAs *Great Compassion*, *Lion Mind*, and *Gratitude* provided by the app were all associated with transitions from the red (HELP) emotional state quadrant to a pleasant emotional state quadrant (green or yellow), whereas *Falling Asleep, Kindness,* and *Commonality of Suffering* were associated with staying in a negative emotional state. For users whose initial emotion was in the blue (LELP) quadrant, the MMAs *Kindness*, *Great Compassion*, and *Counting Breaths* were associated with emotions transitioning out of the green (LEHP) or yellow (HEHP) quadrants. The session index (a proxy for the number of engagements with the app over time) was also associated with users remaining in, or transitioning to, low pleasantness quadrants. Both age and gender were associated with transitions of different types as well. For example, males were more likely than females to transition from negative states to positive states, and older users were more likely to transition to yellow (HEHP) states. There was not enough data to assess the degree to which MMAs could influence users who started in a yellow (HEHP) quadrant and ended in a red (HELP) quadrant.

### Simulating a Learning System

**Figure 9:**
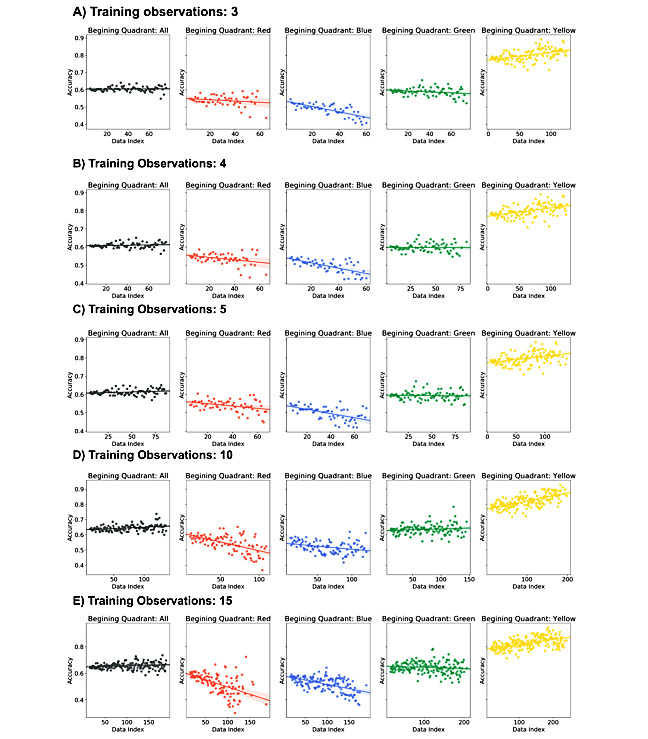
Ending Yale Mood Meter (YMM) quadrant prediction accuracy from distance to training data. Accuracy of models for predicting post-mindfulness and meditation activity (MMA) YMM quadrant emotions with varying number of observations used for training. Accuracy is also shown as a function of the pre-MMA YMM quadrant and suggests differing levels of accuracy and change in accuracy given the pre-MMA quadrant. (A) 3 training observations; (B) 4 training observations; (C) 5 training observations; (D) 5 training observations; (E) 15 training observations.

The most common YMM quadrant that users transitioned to was green, as expected for an app designed to guide users to a calm and meditative state. The green emotional state quadrant accounted for approximately 45% of all emotional states that users transitioned to. Using data associated with the first 3 engagements with an SBT MMA, we built predictive models to determine which states users are most likely to transition to in subsequent engagements. We then compared the predicted transitions with the actual transitions. We found that the predictions were 61% accurate. Subsequent analyses involving the use of more data from users showed consistency with this level of accuracy (Figure 9 and Multimedia Appendix 4). We found that the accuracy of the predictions differed as a function of the quadrant that the emotions were assigned to before engaging in an MMA, with blue (LELP) being the least accurate and yellow (HEHP) being the most accurate. We also found that the more observations used in training the models, the more accurate the predictions become, as expected. If building models like these was pursued with data collected going forward, then the larger sample size would result in greater power to make predictions and hence generate more accurate predictions, thus potentially continually evolving toward a better recommendation for MMAs given an individual’s current mood.

## Discussion

### Data Set

With regard to the clustering of emotions, due to the deidentification process of users, it is difficult to directly compare and synthesize aspects of our current analyses with the results of our previously published studies. Despite this, we noted that, in aggregate, the proportions of users in different emotional state categories were more or less the same (age, gender, and emotion endorsement) between the studies. Our clustering analyses produced similar results, which we interpret as consistency in the underlying data as well as in the final results. The key finding of the previous analysis, that users’ baseline emotional state improved with the number of engagements with the app, helps put into perspective our findings from our most recent analyses.

### Principal Findings

Our analyses suggest that individual MMAs provided by the SBT app have varying degrees of association or potential influence on transitions between emotional states, based on a user’s baseline (pre-MMA) emotional state. Furthermore, we found that there is no reason to believe that a “one-size-fits-all” approach to providing a very general MMA recommendation after observing a user’s poor mood would be beneficial, as not all MMAs affect individuals in the same way. Rather, we found that depending on what a user’s initial mood or mental state was at the time of engagement with the SBT app before pursuing an MMA can influence whether a specific MMA will improve their mental state. Taking this into account, for the app to be more effective, it would be important to “nudge” users away from certain MMAs, which might increase the probability of them remaining in a negative mental state. Gender differences also seem to play a role in how a user’s mood will transition after engaging in an MMA, as males appear to have an easier transition from unpleasant emotional states. The user’s age also seemed to affect how they transition from poor mental states, as older users seem to heavily favor a more pleasant, energetic state.

Ultimately, some clear themes emerged from our analyses focused on each of our 8 mental state clusters. The first PCoA resulting from our cluster analysis most strongly resembles the pleasantness axis of the YMM (Figure 3), with the left-hand side being more pleasant clusters (grateful, calm, compassion, and excited). The second PCoA somewhat resembles the energy axis of the YMM, with the clusters on the top suggesting higher energy (excited, grateful, and angry). However, we found that the anxiety cluster (mapped to red in the YMM; ie, HELP) was more ambiguous. This could suggest that an additional emotional factor underlying anxiety exists that makes it different from other HELP emotions. Additional projections with the third and fourth PCoAs suggest that the angry and anxiety clusters only share a weak similarity, respectively.

When we examined which MMAs drive emotional transitions, we found some common themes as well as a few surprising results. For example, the *Engaging Your Senses* MMA, which appears to be associated with transitioning users from red (HELP) to green (LEHP), asks a user to tune into each of his or her senses in sequence, observing what they notice without evaluating or judging their experience. The ability to observe one’s thoughts, rather than being fully caught up or entangled in them, has been referred to as “metacognitive awareness” and has been shown to be beneficial in dealing with anxiety and stress. This type of MMA may be ideally suited for helping users transition from specific transitions and is consistent with our results. Another well-suited MMA for transitioning away from red (HELP), *Great Compassion,* involves a 3-step process: (1) recognizing that others are just like you and that they want to experience happiness and avoid pain and suffering; (2) broadening one’s attention to include people or pets that they love, people they do not know, and even people they have difficulty with, and then imagining that they are breathing in pain and suffering, and breathing out positive energy; and (3) calling to mind people who are of service to others in the world and who can inspire you to do the same. *Great Compassion* may have the effect of moving people out of an angry state because of the process of putting oneself in another's shoes and then cultivating “big picture” thinking, that is, looking at the world a little differently, from a broader perspective. The *Gratitude* MMA has a similar perspective and has the impact of reframing and also helps to cultivate big picture thinking, helping to put things into a larger perspective. These 3 activities all have a focus on separating one’s thoughts from the current emotions one is experiencing, and thus are quite likely to lead to similar transitions.

The *Lion Mind* MMA, in contrast, is a quieting activity, using the metaphor of a lion mind versus a dog mind to help take one out of “thought loops” (or ruminating thoughts) that feed anger. It is surprising that the MMA *Kindness* seems ineffective, despite being thought of as the antidote to anger in traditional Tibetan Buddhist philosophy, from which the MMA is derived. It may be that this type of activity works better as a long-term remedy to anger but does not work as an immediate solution that could be used in, for example, anger management strategies. *Commonality of Suffering* and *Change* are similar as they help put things into perspective by tapping into your empathy. With a broader perspective, it is supposed to be easier to feel more relaxed about your own situation or feelings, but users may not be able to reach this perspective given their emotional state. One additional complication may be that *Kindness* and *Change* use a somewhat different and more traditional way of communicating when compared with other MMAs.

For transitions that start from the blue (LELP) quadrant, we obtained surprising results but still notice some common themes. For MMAs that lead to a favorable transition, for example, *Kindness*, *Great Compassion*, Counting *Breaths*, and *Equanimity*, there is a strong focus on interconnectedness and the development of bonds to others. Using this information to anticipate the need for effecting changes in mental state, one could create an app that can better recommend and understand why some MMAs might be more effective given a user’s emotional state.

Our previous analysis suggested that a user's baseline emotional state improved with continued use of the application. However, in this study, we noticed that the longer the user engaged with the app, the less likely they are to transition from a negative state, as defined by associations with literature-defined mood quadrants. While counterintuitive, these results do not contradict each other. We do see more users both starting and ending in green (LEHP) or yellow (HEHP) states (as shown in the learning system modeling studies). This might suggest that there are some users who use the app but may not find it effective despite long-term use, or there is a gradual “inoculation” of sorts, whereby users do not find as much benefit from the app as they have benefitted maximally at some point in time. Thus, the effect of the app seems to plateau, which could be alleviated by introducing new and possibly more effective MMAs to a user inured to a current set of MMAs. In addition, there is a unique MMA, *Falling Asleep*, which is meant to help users relax and fall asleep. The “tired” cluster can be considered as both a negative or positive cluster depending on the circumstances, and users who select the *Falling Asleep* meditation could be moving to this more ambiguous cluster, driving some of the results that we observed.

We find great potential in using the types of models we built to predict the mood a user will transition to based on their MMA selections and initial mood or mental states. Even with a small number of observations, we were able to accurately predict 60.7% of all transitions (15.6% increase over informed guesses and 35.7% increase over random guesses). These models would be more accurate with more data, which suggests that a real-time learning system could be implemented that improves over time to help guide users to MMAs. This would have better chances of a successful mood-elevating transition. As more data are collected, further refinements to the predictive models could be made, as different covariates could be included in the analysis without the worry of overfitting. Other covariates could include, but are not limited to, environmental factors such as time since the last session, last MMA completed, current weather, political events, or other external events in a user’s life. This implies that these models could be incorporated into the application and, in real time, help provide improved recommendations for MMA to specific users. Using these models, MMAs could be suggested in a prioritized list, which are ranked by likelihood of a favorable emotional state based on the user’s initial state and other features.

### Limitations of the Study

Although our study was conducted with data from many users, each with several engagements with the app over varying periods of time, it is entirely observational and does not include the sorts of controls that define, for example, randomized controlled clinical trials. Our analyses were also limited to data reflecting what the users of the app ultimately chose and disclosed within the app. Given that some MMAs have widely differing effects, this would suggest that there are certain MMAs that are likely better suited for inducing different transitions; this phenomenon should be studied in more tightly controlled settings. It should also be noted that the emotional classifications themselves are experimental, and there are many alternative concepts that may differ from our observations in terms of the YMM quadrants [30].

Our filtering criteria for identifying individuals who are appropriate for our analyses could have also created biases in our results. As we examined individuals with 10 or more uses of the app, our attention was naturally confined to individuals who are engaged users and found some personal benefit for its continued use. In contrast, a user who stops after a few uses may not see the same benefits from MMAs in their short experience and hence do not necessarily follow the observed transitions that long-term users exhibit. As noted in our previous publication, long-term use of the app influences basal emotional state. Finally, most users reported being in a YMM green state (LEHP) when engaging with the app initially and did not change their state post-MMA, reducing the number of transitions away from initially poor emotional states that we could study.

### Future Directions

As we confined our attention to specific users (eg, iOS users) and MMAs (ie, only those most widely used), we could expand our analyses to all users and MMAs, possibly by clustering the broader set of MMAs in some way. We focused our analysis on the transitions from initial emotional states based on the chosen MMA but ignored other data that were collected (eg, physical state of the person, sex, geolocation, etc). Therefore, we could assess the degree to which these other factors impact our results. For example, we observed that males generally transition to improved mood more frequently than females (ie, starting in a red state [HELP], males are 29% less likely to transition to blue [LELP] and 20% more likely to transition to green [LEHP]), but we did not test which MMAs work better for males (or females) individually. Knowing the effect of these factors and how similar users react to MMAs could help push our efforts toward truly personalizing features of the app and inducing a desired state of mind in a variety of contexts that may help mitigate or avoid associated mental health concerns or symptoms. Ultimately, we believe our findings could motivate more focused studies, through randomized clinical trials, that could lead to further insights into the effectiveness of apps designed to improve mood and help with mental health disorders.

## Appendix

Multimedia Appendix 1 Descriptions and frequencies of the top 20 mindfulness and meditation activities (MMAs) completed. The remaining MMAs are aggregated as "other.".

Multimedia Appendix 2 Detailed study population statistics broken down by gender.

Multimedia Appendix 3 All 115 endorsable emotions grouped by cluster and associated Yale Mood Meter quadrant.

Multimedia Appendix 4 Predictive accuracy by amount of training data used. The percentage of users who ended up in a given Yale Mood Meter quadrant compared with the prediction accuracy by increasing the amount of training data used.

## Abbreviations

GLM: generalized linear model
HEHP: high energy, high pleasantness
HELP: high energy, low pleasantness
LEHP: low energy, high pleasantness
LELP: low energy, low pleasantness
MMA: mindfulness and meditation activity
PAM: partitioning around medoids
PCoA: principal coordinate analysis
SBT: Stop, Breathe & Think
YMM: Yale Mood Meter
